# Analysis of Complex Mixtures by Chemosensing NMR Using *para*-Hydrogen-Induced Hyperpolarization

**DOI:** 10.1021/acs.accounts.1c00796

**Published:** 2022-06-16

**Authors:** Roan Fraser, Floris P. J. T. Rutjes, Martin C. Feiters, Marco Tessari

**Affiliations:** Institute for Molecules and Materials, Radboud University, Heyendaalseweg 135, 6525 AJ Nijmegen, The Netherlands

## Abstract

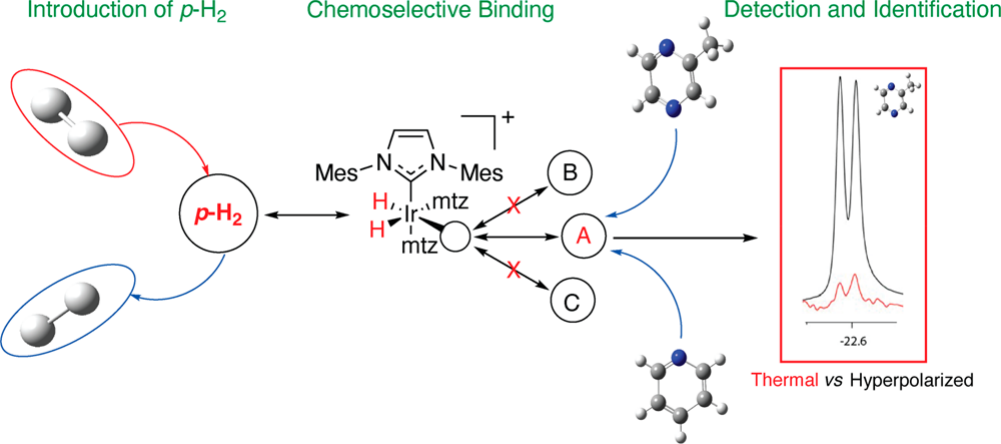

Nuclear magnetic resonance (NMR) is a powerful technique for chemical analysis. The use of NMR to investigate dilute analytes in complex systems is, however, hampered by its relatively low sensitivity. An additional obstacle is represented by the NMR signal overlap. Because solutes in a complex mixture are usually not isotopically labeled, NMR studies are often limited to ^1^H measurements, which, because of the modest dispersion of the ^1^H resonances (typically ∼10 ppm), can result in challenging signal crowding. The low NMR sensitivity issue can be alleviated by nuclear spin hyperpolarization (i.e., transiently increasing the differences in nuclear spin populations), which determines large NMR signal enhancements. This has been demonstrated for hyperpolarization methods such as dynamic nuclear polarization, spin-exchange optical pumping and *para*-hydrogen-induced polarization (PHIP). In particular, PHIP has grown into a fast, efficient, and versatile technique since the recent discovery of non-hydrogenative routes to achieve nuclear spin hyperpolarization.

For instance, signal amplification by reversible exchange (SABRE) can generate proton as well as heteronuclear spin hyperpolarization in a few seconds in compounds that are able to transiently bind to an iridium catalyst in the presence of *para*-hydrogen in solution. The hyperpolarization transfer catalyst acts as a chemosensor in the sense that it is selective for analytes that can coordinate to the metal center, such as nitrogen-containing aromatic heterocycles, sulfur heteroaromatic compounds, nitriles, Schiff bases, diaziridines, carboxylic acids, and amines. We have demonstrated that the signal enhancement achieved by SABRE allows rapid NMR detection and quantification of a mixture of substrates down to low-micromolar concentration. Furthermore, in the transient complex, the spin configuration of *p*-H_2_ can be easily converted to spin hyperpolarization to produce up to 1000-fold enhanced NMR hydride signals. Because the hydrides’ chemical shifts are highly sensitive to the structure of the analyte associating with the iridium complex, they can be employed as hyperpolarized “probes” to signal the presence of specific compounds in the mixture. This indirect detection of the analytes in solution provides important benefits in the case of complex systems, as hydrides resonate in a region of the ^1^H spectrum (at ca. −20 ppm) that is generally signal-free. The enhanced sensitivity provided by non-hydrogenative PHIP (nhPHIP), together with the absence of interference from the complex matrix (usually resonating between 0 and 10 ppm), set the detection limit for this NMR chemosensor down to sub-μM concentrations, approximately 3 orders of magnitude lower than for conventional NMR. This nhPHIP approach represents, therefore, a powerful tool for NMR analysis of dilute substrates in complex mixtures as it addresses at once the issues of signal crowding and NMR sensitivity. Importantly, being performed at high field inside the NMR spectrometer, the method allows for rapid acquisition of multiple scans, multidimensional hyperpolarized NMR spectra, in a fashion comparable to that of standard NMR measurements.

In this Account, we focus on our chemosensing NMR technology, detailing its principles, advantages, and limitations and presenting a number of applications to real systems such as biofluids, beverages, and natural extracts.

## Key References

- EshuisN.; HermkensN.; van WeerdenburgB. J. A.; FeitersM. C.; RutjesF. P. J. T.; WijmengaS. S.; TessariM.Toward nanomolar detection by NMR through SABRE hyperpolarization. J. Am. Chem. Soc.2014, 136, 2695–269810.1021/ja412994k24475903.^1^*The addition of a co-substrate to the catalyst–analyte mixture circumvents the formation of inactive catalyst–solvent complexes and allows for the analysis of analytes in dilute mixtures.*
- EshuisN.; AspersR. L. E. G.; van WeerdenburgB. J. A.; FeitersM. C.; RutjesF. P. J. T.; WijmengaS. S.; TessariM.2D NMR Trace Analysis by Continuous Hyperpolarization at High Magnetic FieldAngew. Chem., Int. Ed.2015, 54, 14527–1453010.1002/anie.20150783126437608.^2^*The continuous hyperpolarization at high magnetic field for the acquisition of 2D NMR measurements of complex mixtures in a submicromolar concentration system has been explored in this study.*
- ReileI.; EshuisN.; HermkensN. K. J.; van WeerdenburgB. J. A.; FeitersM. C.; RutjesF. P. J. T.; TessariM.NMR detection in biofluid extracts at sub-muM concentrations via para-H_2_ induced hyperpolarization. Analyst2016, 141, 4001–400510.1039/C6AN00804F27221513.^3^*PHIP NMR was applied in the study of biofluid extracts (urine in this article)at sub-μM concentrations*.
- SelliesL.; ReileI.; AspersR. L. E. G.; FeitersM. C.; RutjesF. P. J. T.; TessariM.Parahydrogen induced hyperpolarization provides a tool for NMR metabolomics at nanomolar concentrations. Chem. Commun.2019, 55, 7235–723810.1039/C9CC02186H31165813.^4^*PHIP chemosensing was applied for the detection and quantification of specific classes of submicromolar analytes, in complex samples containing hundreds of target analytes.*

## 1 Introduction

### 1.1 *para*-Hydrogen-Induced Polarization

Nuclear magnetic resonance (NMR) is one of the most versatile and powerful analytical methods for the routine analysis of structural and chemical properties of compounds. However, NMR studies of dilute solutions suffer from the relatively low sensitivity of the technique, with a detection limit in the micromolar concentration range. This limitation can be alleviated by nuclear spin hyperpolarization, which provides transiently increased spin population differences, resulting in large NMR signal enhancements. For high-resolution NMR studies, the source of nuclear spin hyperpolarization is mostly a radical excited by microwave radiation, as in dissolution dynamic nuclear polarization (dissolution-DNP)^5−7^ or hydrogen that is enriched in the *para*-spin isomer, *para*-hydrogen (*p*-H_2_), as in *p*-H_2_-induced hyperpolarization (PHIP). Since its discovery,^8^ PHIP has become a recognized approach for signal amplification in NMR spectroscopy,^9^ both with (hydrogenative) and without (non-hydrogenative) incorporation of the atoms of *p*-H_2_ in the analyte. The best-known example of the latter is signal amplification by reversible exchange (SABRE),^9−14^ discovered by Duckett and co-workers^10,12^ in studies with Crabtree’s hydrogenation catalyst [Ir(COD)(PCy_3_)(Py)]^+^, where hyperpolarization could be transferred from *p*-H_2_ to the ^1^H, ^13^C, and ^15^N nuclei in pyridine. SABRE hyperpolarization does not require any unsaturation amenable to hydrogenation, and is therefore achieved without any chemical modification of the analyte (or substrate), which represents an advantage over hydrogenative PHIP. However, analyte coordination to the iridium is required, which still implies some degree of selectivity so that the hyperpolarization catalyst should be considered a chemosensor. In addition to nitrogen heteroaromatics, compound classes that have been SABRE hyperpolarized, with iridium complexes of PCy_3_ (tricyclohexylphosphine)_3_ or other ligands, include amines,^15^ nitriles,^16^ Schiff bases,^17^ diaziridines,^18,19^ and sulfur-containing heterocycles.^20^

**Figure 1:**
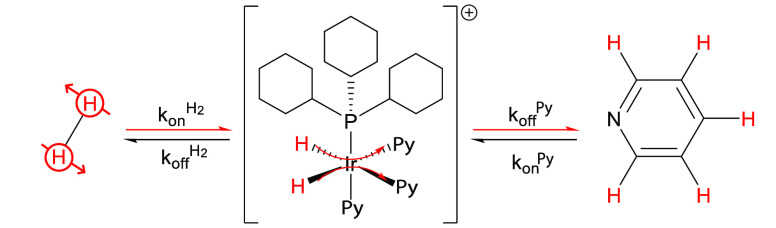
Schematic representation of the SABRE experiment: at low magnetic field, spontaneous transfer of spin order from the hydrides originating from *p*-H_2_ to the nuclear spins of the analyte (substrate) occurs via the scalar coupling network within the transient complex [Ir(PCy_3_)(H)_2_(Py)_3_]Cl, where PCy_3_ and Py refer to tricyclohexylphosphine and pyridine, respectively. The subsequent dissociation of the analyte produces hyperpolarized molecules in solution that can be detected by NMR with enhanced sensitivity. Adapted with permission from ref (1). Copyright 2014 American Chemical Society.

At low field, spontaneous polarization transfer occurs between the hydrides originating from *p*-H_2_ and the nuclear spins of the analyte that is temporarily associated with the iridium catalyst. Continuous ligand association/dissociation leads to the buildup of hyperpolarization of free analyte in solution, which can be detected by NMR after transferring the sample to high magnetic field. The hyperpolarization transfer involves only the ligands in the equatorial plane (Figure 1), with a nonvanishing scalar coupling to the hydrides and association/dissociation rates comparable/faster than the NMR relaxation rate of the hydrides. Conversely, the nuclear spins of the analyte in the *cis* position with respect to the PCy_3_ ligand are not directly hyperpolarized because of the absence of scalar couplings with the hydrides and much slower dissociation kinetics.

Figure 1 summarizes the most important kinetic processes influencing SABRE efficiency. Studies of the dependence of SABRE on ligand concentration have shed light on the mechanism of *p*-H_2_ refreshment in the complex, which is crucial for an efficient hyperpolarization. The first step in this process is the dissociation of a substrate unit from the equatorial plane of the complex, with the formation of a 16-electron intermediate [Ir(PCy_3_)(H)_2_(Py)_2_]^+^ that is necessary for the subsequent association/dissociation of *p*-H_2_. The analyte exchange rates are controlled by steric and electronic factors; the sterically bulky PCy_3_ has been replaced by stronger electron-donating NHC (*N*-heterocyclic carbene) ligands,^11^ for which systematic investigation^21,22^ has shown that the pyridine exchange rate increases with buried volume (%*V*_bur_),^23^ with optimal SABRE hyperpolarization observed for the IMes ligand.

This Account focuses on the application of non-hydrogenative PHIP in our chemosensing NMR technology^24^ to selectively detect and quantify specific analytes (e.g., nitrogenous heteroaromatic compounds) in solution at down to nanomolar concentrations (i.e., 1000 times more dilute than for conventional NMR).

## 2 Analysis of Dilute Solutions with SABRE/nhPHIP

### 2.1 Co-substrate Approach

**Figure 2:**
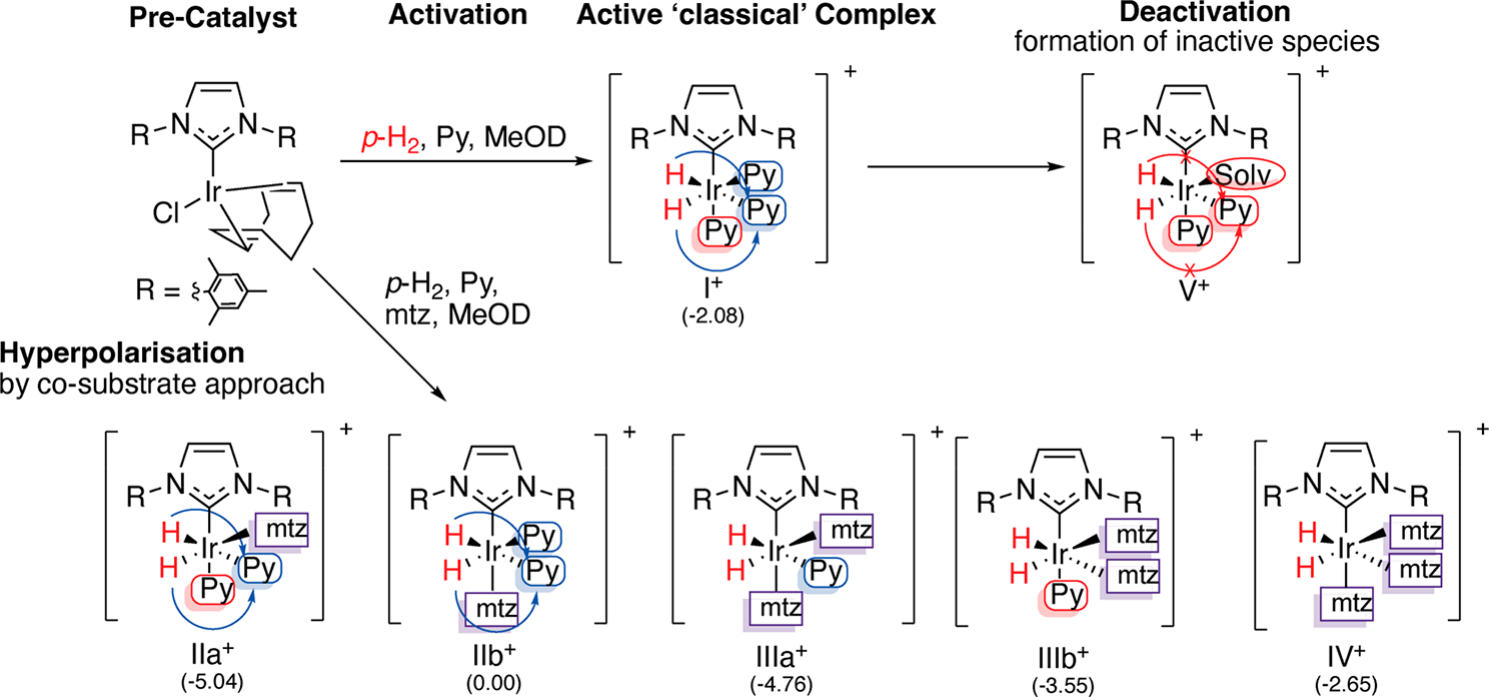
Coordination arrangements possible with analyte pyridine and co-substrate methyltriazole; relative calculated energies are indicated in parentheses (kcal mol^–1^). Adapted with permission from ref (25). Copyright 2015 Wiley-VCH.

SABRE can quickly produce high levels of spin hyperpolarization when performed in the presence of a large excess of analyte with respect to the catalyst concentration so that complex I^+^ (Figure 2, top middle) in which hyperpolarization is transferred predominates in solution. Conversely, the application of SABRE to dilute substrates results in negligible NMR signal enhancements. This is probably due to the formation of unstable asymmetric complexes such as V^+^ (Figure 2, top right), involving solvent molecules as ligands, resulting in the fast conversion of *para*-enriched to thermal H_2_ as well as an inefficient polarization transfer to the nuclear spins of the analyte.

We have demonstrated that SABRE/nhPHIP hyperpolarization at low analyte concentration can be restored by the addition of a concentrated ligand (a so-called co-substrate):^1^ in the presence of an excess of 1-methyl-1,2,3-triazole (mtz), NMR detection of pyridine at 1 μM was achieved in a single scan via SABRE hyperpolarization. The co-substrate mtz binds to the iridium catalyst with higher affinity than the solvent molecules; therefore, if present at a higher concentration than the catalyst precursor, it minimizes the formation of unstable complexes that are detrimental to SABRE/nhPHIP. As demonstrated by DFT calculations, the association of the pyridine analyte to form the mixed complexes [Ir(IMes)(H)_2_(mtz)(analyte)_2_]Cl (IIa) and [Ir(IMes)(H)_2_(mtz)_2_(analyte)]Cl (III) is energetically favored compared to the pure mtz complex IV (Figure 2, bottom).^25^ A crucial factor for SABRE/nhPHIP is the preference of the [Ir(IMes)(H)_2_(mtz)_*x*_(analyte)_3–*x*_]Cl complexes for an asymmetric configuration in the equatorial plane such as in complexes IIa and IIIa. This means that the analyte occupies a *cis* position, in the equatorial plane, so that both conditions for effective SABRE, viz., scalar coupling with the hydrides originating from *p*-H_2_ and relatively fast dissociation, are fulfilled.

Co-substrates, often named co-ligands, have also proved useful for the hyperpolarization of weakly coordinating analytes. Examples are the enhancements of the hyperpolarization of acetonitrile in methanol with Ir-IMes, with or without an additional PCy_3_ ligand, by pyridine,^26^ of amines by acetonitrile or mtz,^27^ of *o*-NH_2_-substituted pyridines and pyrimidines by acetonitrile or allylamine,^15^ and of pyruvate^28^ and ketoisocaproate^29^ by dimethyl sulfoxide.

**Figure 3:**
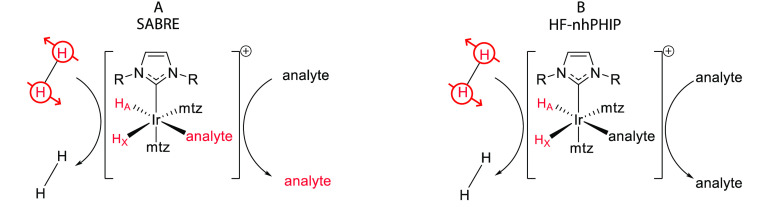
(A) Schematic representation of the SABRE experiment at low magnetic field: spontaneous transfer of spin order from the hydrides originating from *p*-H_2_ to the nuclear spins of the analyte occurs via the scalar coupling network within the transient complex [Ir(IMes)(H)_2_(analyte)(mtz)_2_]Cl. Subsequent dissociation of the analyte produces hyperpolarized molecules in solution to be detected by NMR with enhanced sensitivity. (B) Schematic representation of reversible PHIP at high magnetic field: formation of the asymmetric complex [Ir(IMes)(H)_2_(analyte)(mtz)_2_]Cl due to the reversible association of *p*-H_2_ and analytes determines the hyperpolarization of the hydrides, which can be revealed by up to 1000-fold enhanced NMR signals. mtz, methyl-1,2,3-triazole. Adapted with permission from ref (1). Copyright 2014 American Chemical Society.

Figure 3 illustrates two different reversible PHIP approaches, based on the addition of mtz as a co-substrate, that make use of the very same catalytic PHIP machinery but are fundamentally different in their implementation as well as in their strengths and weaknesses, as will be discussed in the following paragraphs.

### 2.2 SABRE

SABRE involves the aforementioned spontaneous conversion of the singlet spin order of the hydrides to enhanced magnetization of the analyte at low magnetic field. After transferring the sample to high magnetic field, NMR detection down to submicromolar concentrations in the presence of the mtz co-substrate (Figure 3A) is possible. The SABRE experiment provides hyperpolarized free analyte in solution that can be easily identified by a simple chemical shift comparison with an NMR database. For highly dilute analytes, the signal-to-noise ratio can be improved by signal averaging and acquiring multiple SABRE experiments. To achieve reproducible results, a flow system has been developed to automatically shuttle the sample back and forth between a low-field chamber (where polarization transfer to the analytes occurs) and high field for NMR detection (see Section 3.1).^30^

### 2.3 High-Field Non-Hydrogenative PHIP (HF-nhPHIP)

The PHIP approach (Figure 3B) is more accurately referred to as high-field non-hydrogenative PHIP (HF-nhPHIP) to distinguish it from classical PHIP. PHIP in the broader sense also features hydrogenation and non-hydrogenative techniques such as SABRE. HF-nhPHIP focuses on the complex resulting from analyte association to the iridium catalyst rather than the free form in solution and is achieved directly at high magnetic field (i.e., inside the NMR magnet). In the presence of *p*-H_2_ and excess co-substrate mtz, dilute analytes in solution (anal_*i*_) transiently associate with the hyperpolarization catalyst, predominantly forming the complex [Ir(IMes)(H)_2_(mtz)_2_(anal_i_)]Cl. The formation of mixed-analyte complexes [Ir(IMes)(H)_2_(mtz)(anal_*i*_)(anal_*j*_)]Cl is negligible because of the large excess (typically 3 to 4 orders of magnitude) of the co-substrate. The asymmetric configuration in which both the analyte and mtz occupy the equatorial plane is energetically favored (see Section 2.1), which is crucial for nhPHIP/SABRE hyperpolarization. The hydrides in such asymmetric complexes are not chemically equivalent, which causes the rapid dephasing of the singlet state to longitudinal spin order. A SEPP (selective excitation of polarization using PASADENA)^31,32^ pulse scheme allows the conversion of this spin order to enhanced magnetization and, subsequently, NMR detection of two hyperpolarized hydride signals for each analyte associating with the iridium complex. Importantly, the chemical shifts of these hydrides are highly sensitive to the structure of the corresponding analyte and can therefore be employed as hyperpolarized “probes” to signal the presence of specific compounds in the sample.^33^

The catalyst acts in this respect as an nhPHIP-NMR chemosensor, signaling the presence of specific analytes in solution via enhanced hydride signals. Importantly, the response of this NMR chemosensor does not suffer from the (sometimes large) contributions from the sample matrix because hydrides resonate in a region of the ^1^H spectrum (at ca. −20 ppm) that is generally signal-free. The enhanced sensitivity/specificity and background removal make this nhPHIP-NMR chemosensor highly suitable for the investigation of dilute components in complex mixtures, such as natural extracts and biofluids. Spectral resolution of overlapping signals can be achieved by 2D nhPHIP-NMR experiments correlating the hydride resonances. Alternatively, the spin order of the hydrides can be used to acquire enhanced 2D correlation spectra with the analyte protons via long-range scalar couplings in the complex.^2^ A clear advantage of this high-field setting is that hyperpolarization can be realized in a continuous fashion at the beginning of each transient, by briefly (typically 1–3 s) bubbling *p*-H_2_ in the NMR tube inside the spectrometer. This allows the combination of nhPHIP with signal averaging for a further sensitivity increase as well as the implementation of standard NMR tools (phase cycling, multidimensional experiments, etc.).

Because the chemical shifts of the hydrides in the complex are the only source of information about a compound associating with the catalyst, structure identification of analytes via nhPHIP is far more cumbersome than for SABRE and is generally performed by trial and error, spiking the solution with pure compounds. However, the hydrides’ chemical shifts are highly sensitive to the structure of the analyte associating with the iridium complex and reproducible, irrespective of the specific mixture under investigation. Therefore, a database of chemical shifts of hydrides for SABRE/nhPHIP analytes is presently being compiled to assist analyte identification.

### 2.4 Quantitative Analysis of nhPHIP/SABRE Spectra of Dilute Analytes

**Figure 4:**
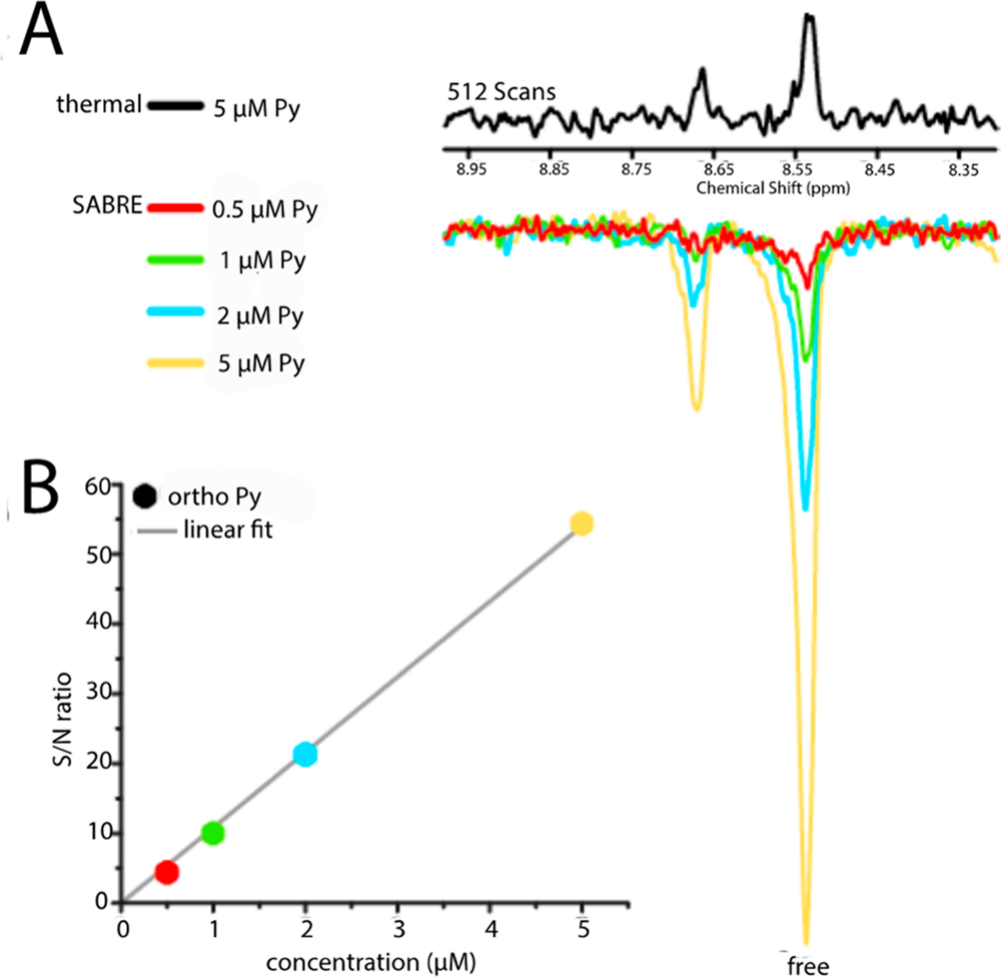
(A) ^1^H NMR signals acquired at 600 MHz, at thermal equilibrium (black) or following SABRE hyperpolarization (colored) of samples containing trace amounts of Py together with complex precursor [Ir(SIMes)(COD)Cl] (SIMes = 1,3-bis(2,4,6-trimethylphenyl)imidazolin-2-ylidene) (1 mM), mtz (18 mM), and 4 bar 51% enriched *p*-H_2_ in methanol-*d*_4_. The displayed signals originate from the *ortho*-protons of Py in the free and bound forms. (B) Signal-to-noise ratio of the free Py signals in A as a function of Py concentration. Adapted with permission from ref (1). Copyright 2014 American Chemical Society.

The assumption of a linear dependence of nuclear magnetization on concentration, as stated by Curie’s law for samples at thermal equilibrium, is generally not correct for hyperpolarized samples. NMR quantification of hyperpolarized analytes in solution is therefore not straightforward. However, under suitable conditions, the integrals of hyperpolarized signals depend linearly on concentration, an essential requirement for quantitative NMR applications. If the concentrations of analyte (anal), co-substrate (cosub), and catalyst precursor (cat) satisfy the condition
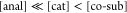
then the distribution of the analyte between free and bound forms is determined by the chemical equilibrium
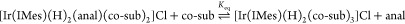
from which eq 1 can be derived:
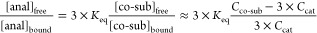
1*K*_eq_ is the relative affinity of the analyte and co-substrate for the metal center, and *C*_cosub_ and *C*_cat_ are the analytical concentrations in a solution of co-substrate and catalyst precursor, respectively. Equation 1 indicates that the distribution between the free and bound forms of a dilute analyte is independent of its total concentration and is determined by the amount of co-substrate and catalyst precursor present in solution. As a consequence, under the aforementioned conditions, a linear dependence of the SABRE/nhPHIP signal on analyte concentration is expected,^1^ as shown for pyridine at low micromolar concentrations in the presence of a large excess of co-substrate mtz (Figure 4B). Therefore, in the low-concentration regime, in which a linear dependence between the SABRE/nhPHIP signal and concentration holds, calibration techniques, such as standard addition,^34^ can be employed to quantify dilute analytes in solution.^33^

Standard addition is performed by increasing the analyte concentration in consecutive steps and acquiring a SABRE or nhPHIP spectrum after each addition. A linear dependence of the signal integral on added analyte concentration is observed. The original analyte concentration is estimated from the abscissa intercept of the standard addition curves (see Section3.2.1.). Quantification by standard addition has also been demonstrated for SABRE-RELAY^35^ on ^13^C in carbohydrates.

## 3 Applications of SABRE/nhPHIP at Low Concentrations

### 3.1 Applications of SABRE to Mixtures of Dilute Analytes

**Figure 5:**
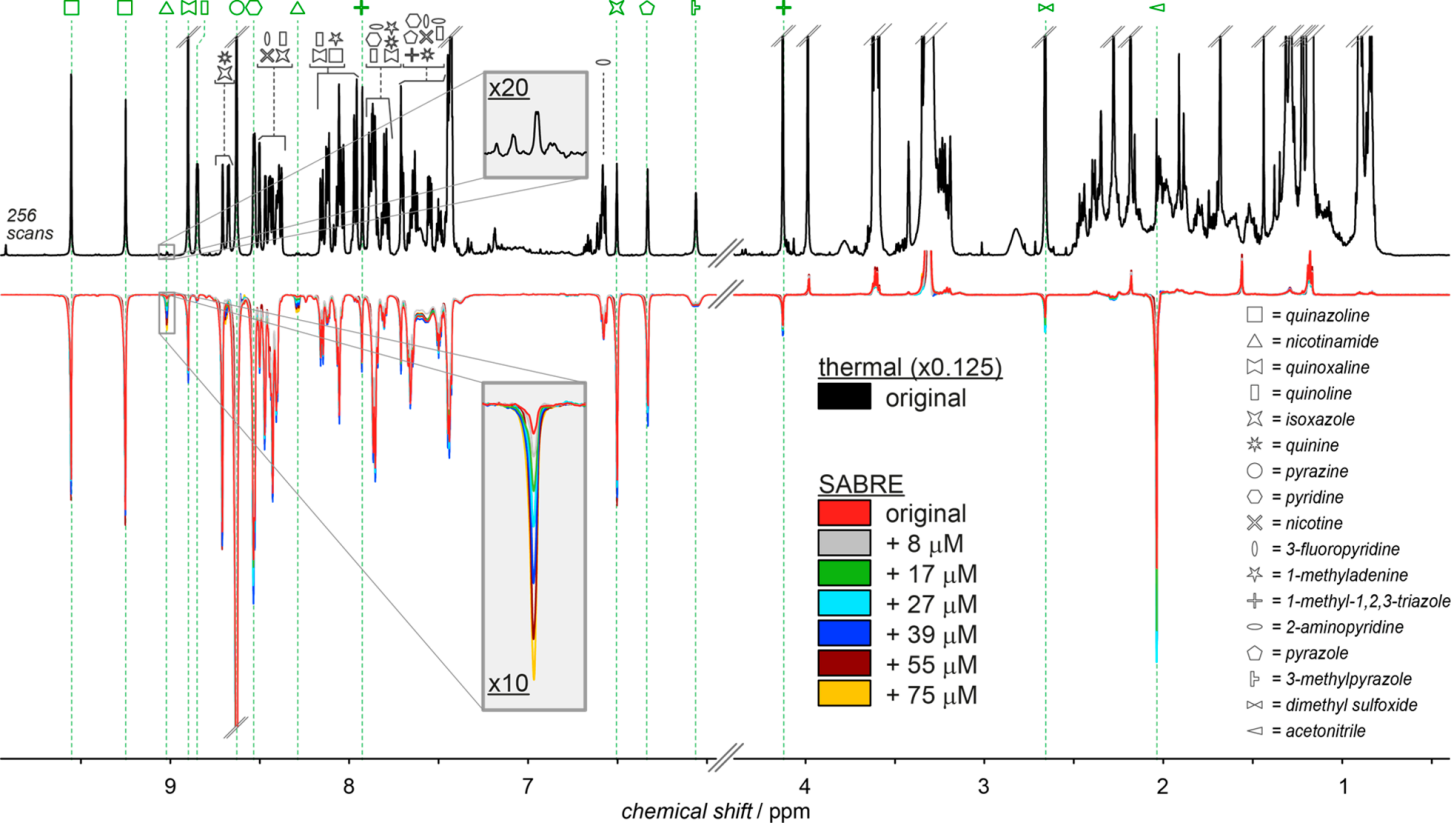
^1^H NMR spectra acquired at 600 MHz, at thermal equilibrium (top, 256 scans, ×0.125 vertically scaled) or after SABRE hyperpolarization at 6.5 mT (bottom, red trace) on a sample consisting of nicotinamide as the analyte (*C*_*x*_ = 8.5 μM), catalyst precursor (*C*_cat_ = 333 μM), and mixtures of 15 high micromolar analytes (*C*_TOT_ = 6 mM). The SABRE spectra of samples after standard addition, with *C*_add_ between 8 and 75 μM (color coded), show increasing nicotinamide signals, while the signals of other analytes remain constant. Insets show one of the nicotinamide *ortho* resonances with a S/N ratio of 31:1 after hyperpolarization (red trace, ×10 vertically scaled) and 15:1 after 256 scans at thermal equilibrium (black trace, ×20 vertically scaled). Adapted with permission from ref (33). Copyright 2015 Wiley-VCH.

SABRE can be successfully applied to dilute analyte mixtures without a co-substrate, provided the total analyte concentration in solution is sufficiently high to saturate the iridium catalyst, preventing solvent association (Figure 5).^33^ The quantification is also in this case performed by standard addition.

**Figure 6:**
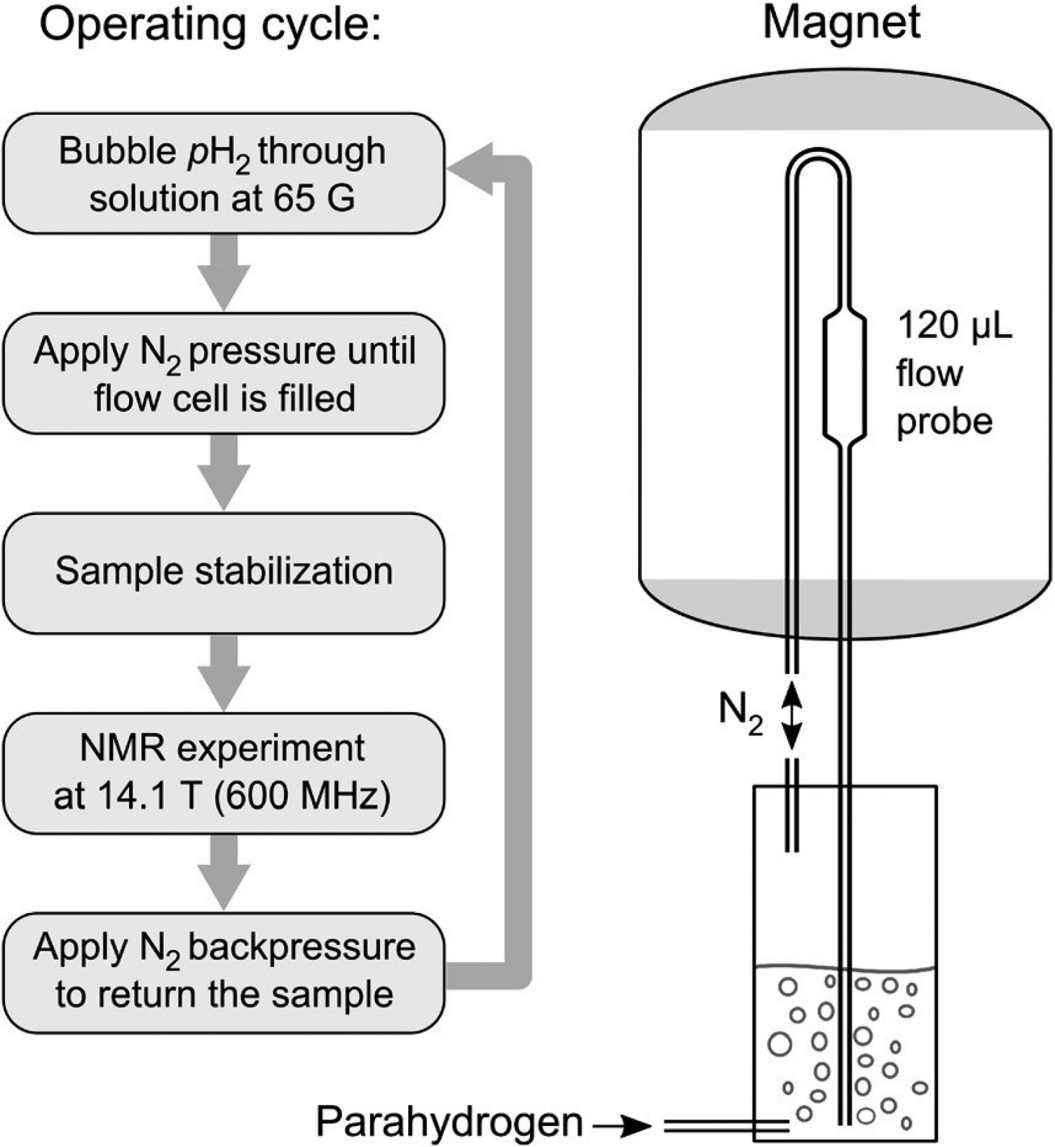
Schematic of the SABRE polarizer. The system provides automated flow control and handles mixing with *p*-H_2_ in a controlled low-field chamber appropriate to SABRE. Adapted with permission from ref (38). Copyright 2017 Wiley-VCH.

Spectral analysis can suffer from extensive signal overlap. Duckett^14^ demonstrated the possibility to extend SABRE hyperpolarization to standard 2D NMR methods, such as 2D COSY and 2D HMBC, typically employed for structure determination of small molecules in solution. This requires the aforementioned (see Section2.2.) flow system^30^ to shuttle the NMR sample back and forth between low and high magnetic field for respectively hyperpolarization and NMR detection (Figure 6).

Alternatively, ultrafast techniques^6^ can be employed in combination with SABRE to acquire single-scan 2D NMR spectra without the requirement of shuttling the sample between low and high magnetic fields multiple times.^36^ With this approach, a single-scan 2D COSY of a mixture of pyridine-like SABRE analytes at submillimolar concentrations was acquired.^36^

**Figure 7:**
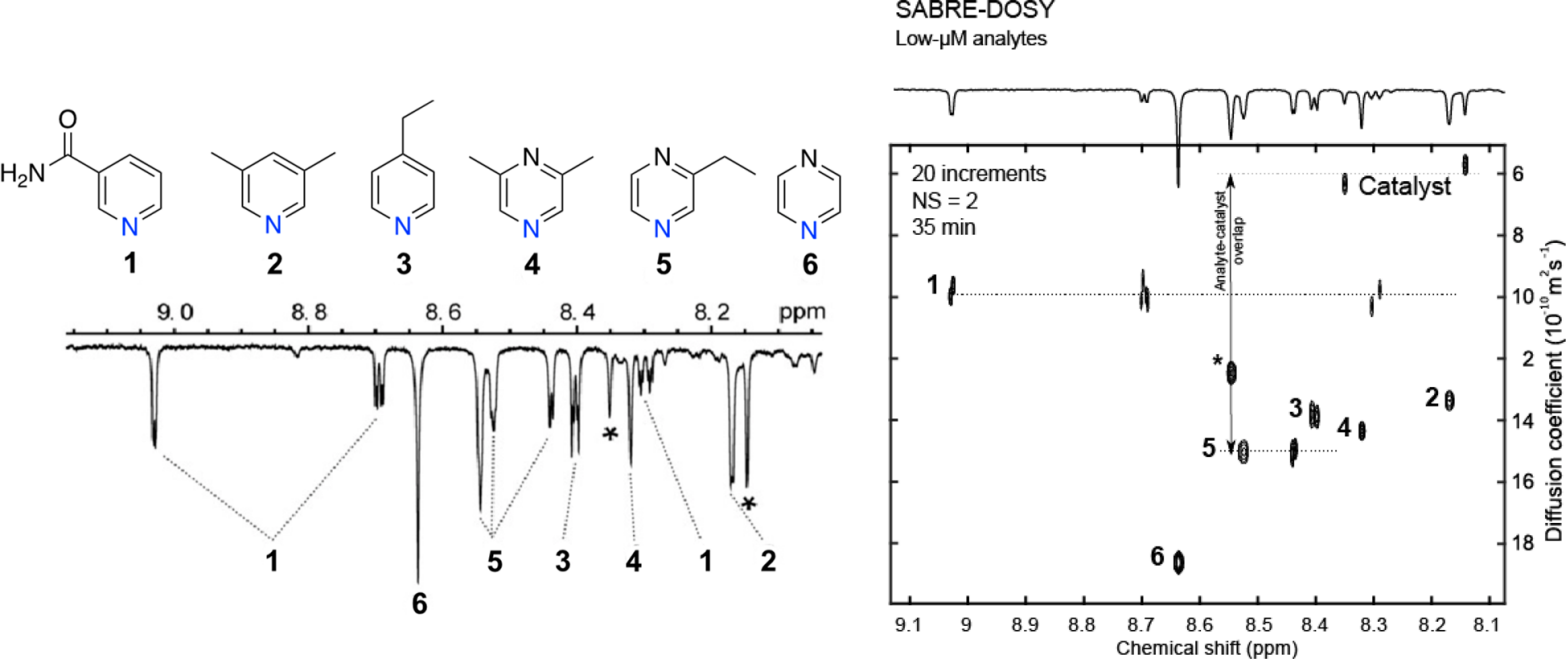
(Left) Region of interest in the SABRE spectrum; analyte concentrations 25 μM (1), 10 μM (2–5), and 5 μM (6). Catalyst-mtz background signals are highlighted with (*). (Right) 2D plot of low-micromolar SABRE-DOSY spectrum in methanol-*d*_4_ recorded in 35 min at 600 MHz. Adapted with permission from ref (38). Copyright 2017 Wiley-VCH.

SABRE has also been successfully combined with diffusion ordered spectroscopy (DOSY),^37^ a popular NMR technique that resolves resonances according to the analyte’s diffusion coefficients, providing a tool to correlate NMR signals and to estimate the number of components in mixtures. While applications of DOSY at low concentrations are generally held back by excessively long measurement times, the enhanced NMR sensitivity provided by SABRE hyperpolarization allows the concentration requirements to be reduced by at least 100-fold. In a SABRE-DOSY spectrum acquired with the aforementioned flow system (Figure 6), signal separation at low micromolar concentration was achieved for a mixture of pyridines and pyrazines with signal enhancements ranging between 90 and 232 and a substantial reduction (∼10^4^-fold) in measuring time (Figure 7).^38^

The suppression of the background signals originating from the sample matrix, which would otherwise hamper the analysis of dilute hyperpolarized compounds, is important for natural extracts. The selection of SABRE-derived signals can be achieved by introducing an only para-hydrogen spectroscopy (OPSY) building block^39^ at the beginning of a pulse sequence; this selects *p*-H_2_-derived hyperpolarized signals through a pulsed field gradients-based coherence filter and removes NMR thermal signals that do not originate from polarization transferred under SABRE.

### 3.2 Applications of nhPHIP to Mixtures of Dilute Analytes

#### 3.2.1 Methanol Extract of Coffee

**Figure 8:**
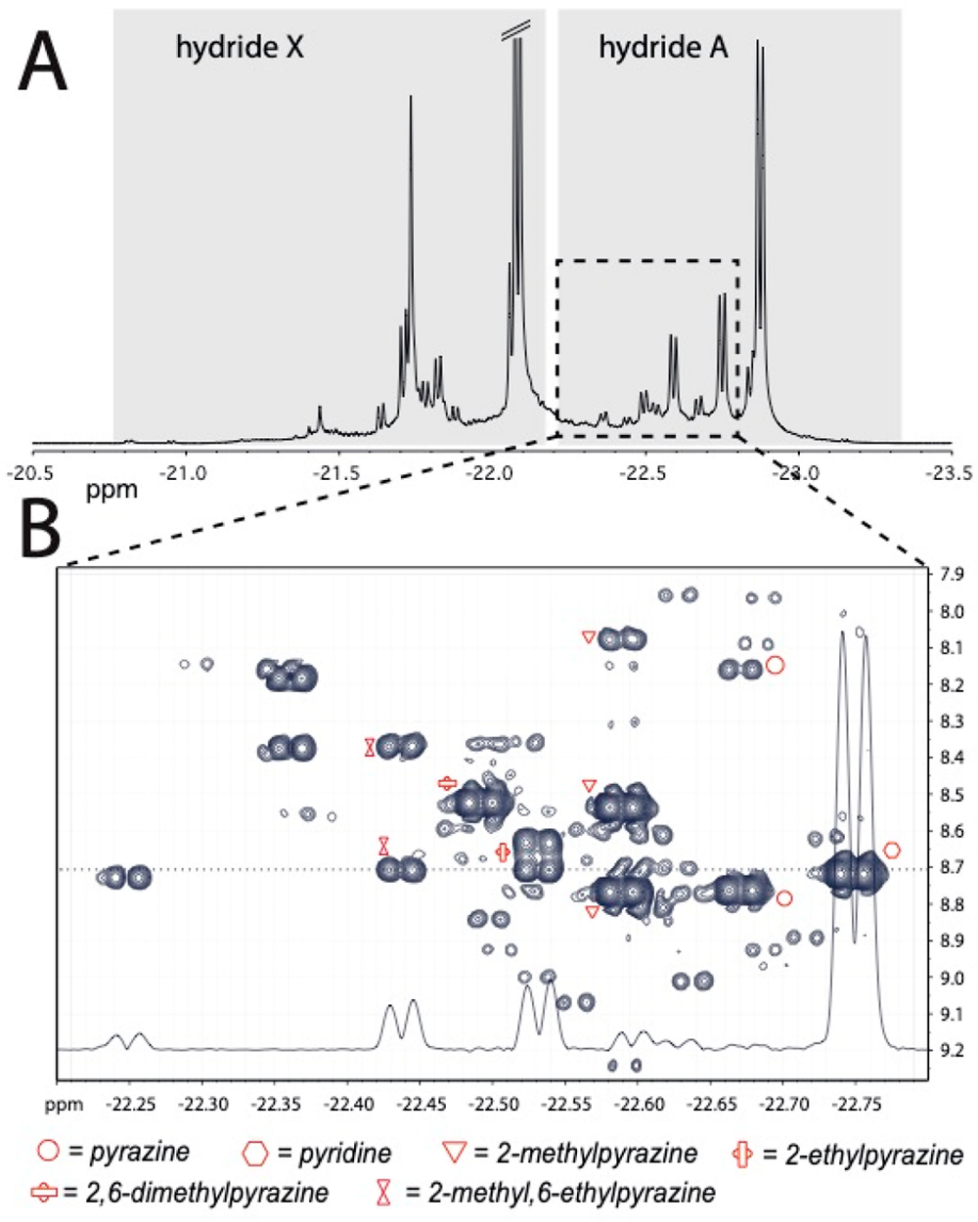
(A) *p*-H_2_ enhanced NMR hydride signals of coffee extract in methanol-*d*_4_, with a 1.2 mM metal complex, 18 mM mtz, and 5 bar 51% enriched *p*-H_2_. (B) High-field *p*-H_2_ enhanced 2D correlation spectrum between hydrides and analyte aromatic protons in the receptor complexes, acquired on the same sample as for A in ca. 20 min. The assignment of the most concentrated species is indicated. The 1D trace is shown to illustrate the signal-to-noise ratio at the dotted line in the spectrum. Adapted with permission from ref (40). Copyright 2016 American Chemical Society.

Because of its selective hyperpolarization, the nhPHIP-NMR-chemosensing approach using HF-nhPHIP (see Section 2.3) provides the opportunity to concurrently detect and differentiate specific classes of compounds, which makes it appropriate for the chemical analysis of complex mixtures without any prior sample fractionation. An example is the quantitative detection of nitrogen heteroaromatics in methanol extracts of ground roasted coffee.^40^ Pyridine, pyrazine, and their derivatives are well known for their occurrence in food products, especially when the processing allows reactions of amino acids alone (Strecker degradation) or amino acids with carbohydrates (Maillard reaction) to take place, as during the roasting of green coffee beans,^41^ resulting in an important contribution to the flavor. Although conventional NMR of a coffee extract is hampered by low concentration and signal crowding, detection via nhPHIP-NMR-chemosensing was straightforward (Figure 8A), with an approximate 10^3^-fold enhancement of the NMR signal response. With iridium-IMes as the catalyst and mtz as the co-substrate, the high-field hydride (“A” in Figure 8A) is typically the one facing the analyte, interacting with analyte protons in the complex via long-range scalar couplings.

**Figure 9:**
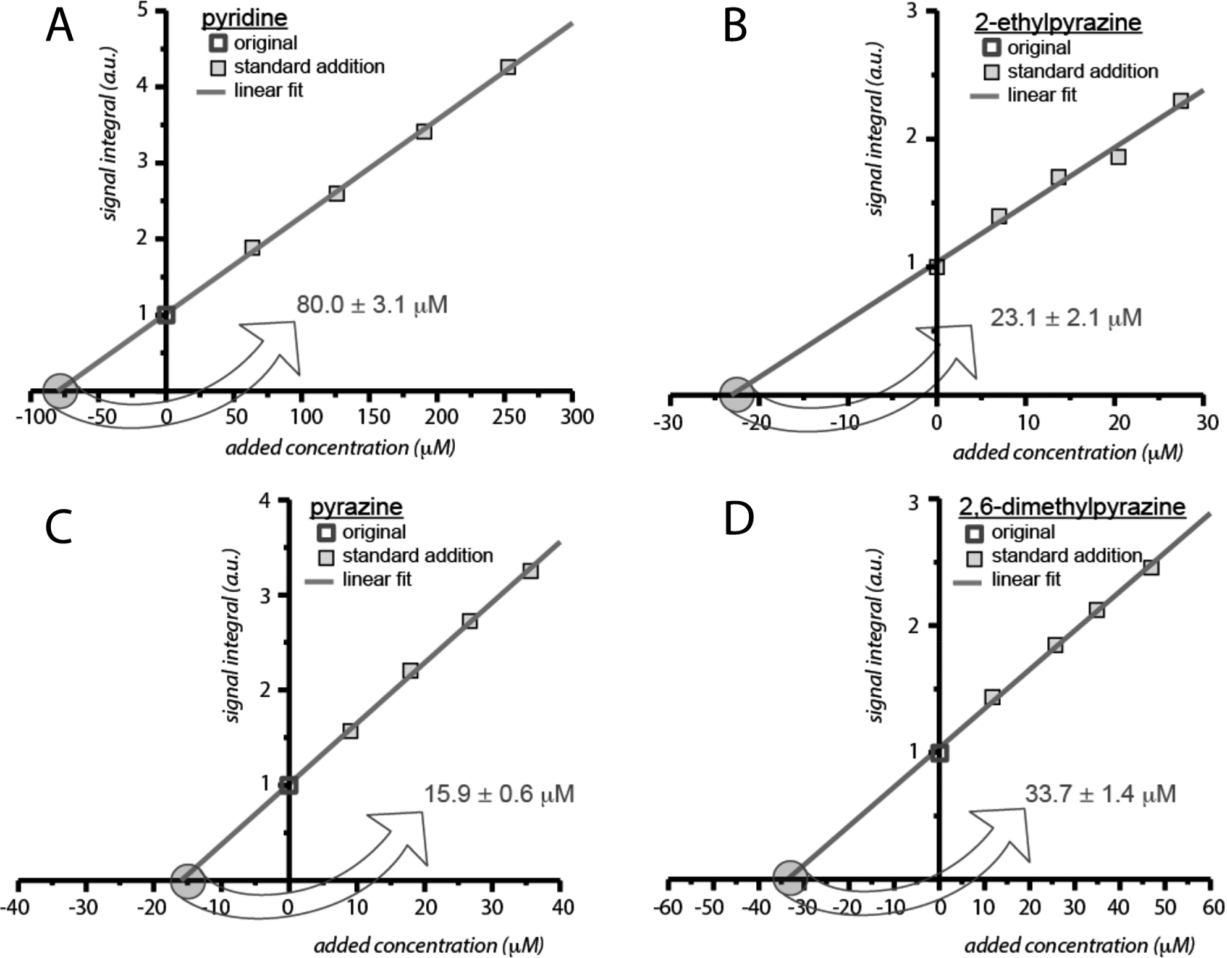
Standard addition curves for *ortho*-proton resonances of pyridine in A, 2-ethylpyrazine in B, pyrazine in C, and 2,6-dimethylpyrazine in D. Concentrations are estimated from the abscissa intercept (circled) of the standard addition curves (gray lines). Experimental uncertainties were derived by error propagation. Adapted with permission from ref (40). Copyright 2016 American Chemical Society.

The signal overlap in the hydride region, due to the large number of compounds in the coffee extract associating with the iridium catalyst, could be resolved by a 2D nhPHIP-NMR correlation spectrum (Figure 8B) between hydrides A and the aromatic protons of the analytes via long-range couplings (*J* ≈ 1.3 Hz, at most).^42^ The assignment of some 2D correlations in the spectrum was obtained by comparison with the nhPHIP-NMR spectra of pure compounds associated with the catalyst. Quantification of the identified components was achieved by standard addition (Figure 9).

#### 3.2.2 Whisky, an Ethanol/Water Mixture

**Figure 10:**
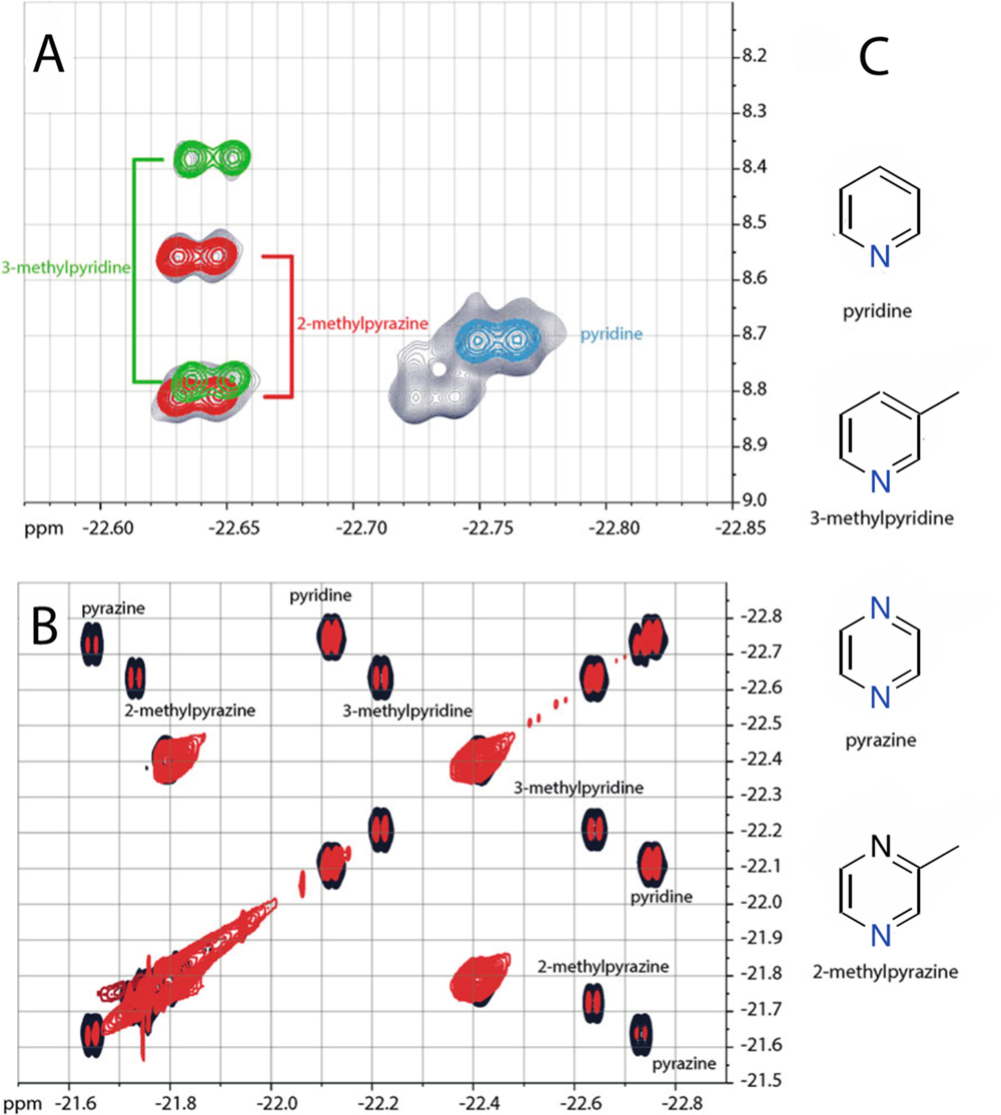
2D NMR spectra of compounds identified in water–alcohol mixtures. (A) Overlay of 2D nhPHIP-NMR correlation spectra between hydrides and *ortho*-aromatic protons in the iridium complex measured on whisky (thin lines, black), whisky spiked with pyridine (cinder, thick lines), and 3-methylpyridine (green) and 2-methylpyrazine (red). (B) Overlay of the 2D nhPHIP DQF-COSY spectrum between hydrides measured on whisky (red) and on whisky spiked with approximately 25 nmol of pyridine, pyrazine, 3-methylpyridine, and 2-methylpyrazine (black). (C) Relevant structures. Adapted with permission from ref (45). Copyright 2018 Wiley-VCH.

Pyrazine and pyridine were also studied by nhPHIP-NMR-chemosensing of an Islay cask strength (58% ethanol/vol) single malt Scotch whisky, for which they are known to produce, respectively, pleasant and unpleasant effects on the taste.^43,44^ Measurement in a methanol–whisky mixture containing ca. 30% water showed a 4-fold decrease in nhPHIP-NMR intensity of the hydride signals compared to a methanol solution,^45^ presumably due to the lower solubility of *p*-H_2_ and the different exchange kinetics of the ligands in water. Nevertheless, four main compounds could be detected and identified in an nhPHIP-NMR 2D spectrum correlating hydrides and aromatic signals (Figure 10a), together with several minor components which remain to be identified.

nhPHIP-NMR 2D COSY spectra correlating the two hydrides gave well-resolved interhydride cross-peaks, of which the linear dependence on analyte concentration was used for the quantitative determination of the four identified analytes via standard addition (Figure 10b). Because this experiment depends on the larger interhydride scalar coupling (7.5–8.5 Hz), it determines a complete and much faster transfer of magnetization than the aforementioned correlation experiment between hydrides and aromatics (see Section3.2.1), providing, therefore, superior sensitivity.

#### 3.2.3 Analysis of Aqueous Mixtures with Solid-Phase Extraction

**Figure 11:**
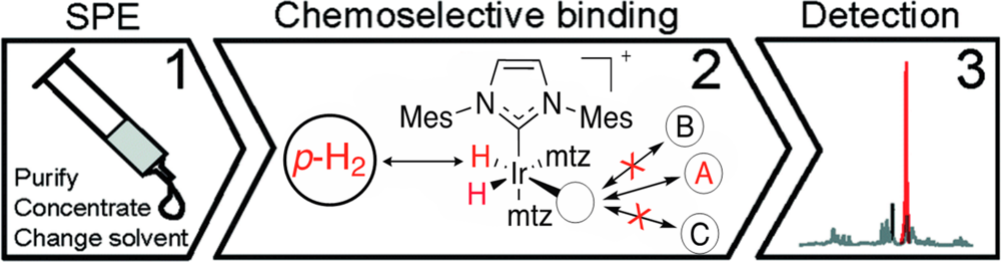
nhPHIP hyperpolarization using SPE for purification, concentration, and solvent change. (1) SPE purification of the analyte mixture, (2) selective hyperpolarization of analyte A (chemosensing), and (3) detection of the chemoselected analyte. Adapted with permission from ref (3). Copyright 2016 the authors. Published by Royal Society of Chemistry under a Creative Commons Attribution-NonCommercial 3.0 Unported License (https://creativecommons.org/licenses/by-nc/3.0/).

Although alternative catalysts that directly allow nhPHIP in aqueous systems have been recently developed,^19,46−49^ their performance is still well below that of the iridium catalyst in methanol. Therefore, the application of nhPHIP-NMR to aqueous mixtures requires sample pretreatment for water removal, which can be straightforwardly implemented by solid phase extraction (SPE) cartridges. These allow water and bulk polar components such as salts to pass, retaining low-polarity compounds that can be subsequently eluted with deuterated methanol for nhPHIP-NMR analysis, as summarized in Figure 11.

**Figure 12:**
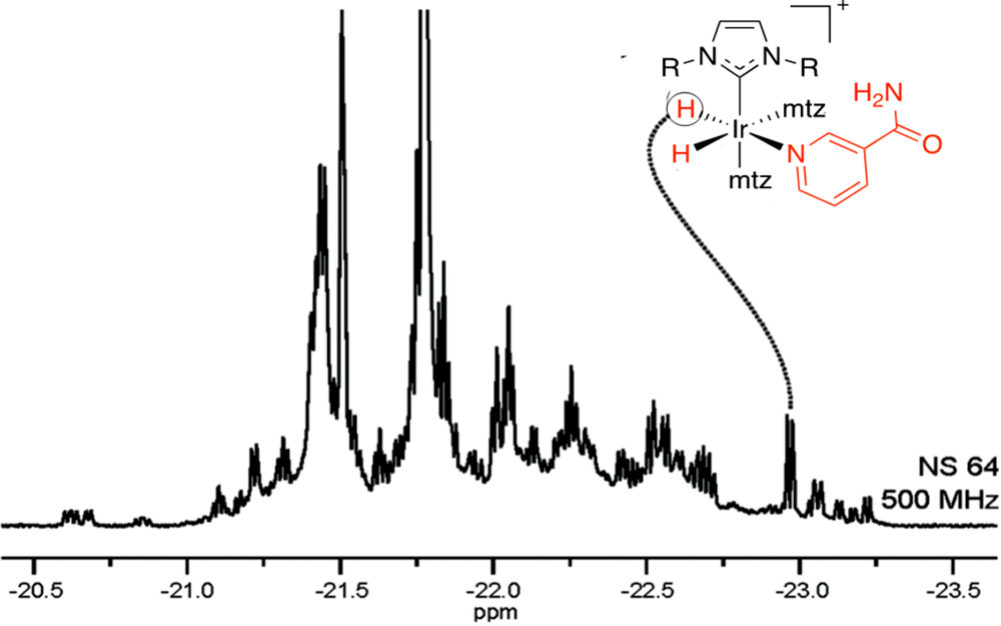
Hyperpolarized hydride signals in SPE of urine using nhPHIP-NMR-chemosensing (51% *p*-H_2_, 1.2 mM iridium catalyst, 18 mM mtz). Adapted with permission from ref (3). Copyright 2016 the authors. Published by Royal Society of Chemistry under a Creative Commons Attribution-NonCommercial 3.0 Unported License (https://creativecommons.org/licenses/by-nc/3.0/).

SPE pretreatment even allows nhPHIP-NMR-chemosensing to be applied for the selective detection of specific classes of metabolites in urine;^3^ this contains more than 4000 compounds in a wide concentration range (∼10^6^), resulting in extensive signal overlap, so that dilute analytes are masked by more abundant components.^50^ Because of the large number of urinary metabolites capable of associating with the iridium catalyst, nhPHIP-NMR-chemosensing of SPE-treated urine produces a highly crowded hydride spectrum (Figure 12).

**Figure 13:**
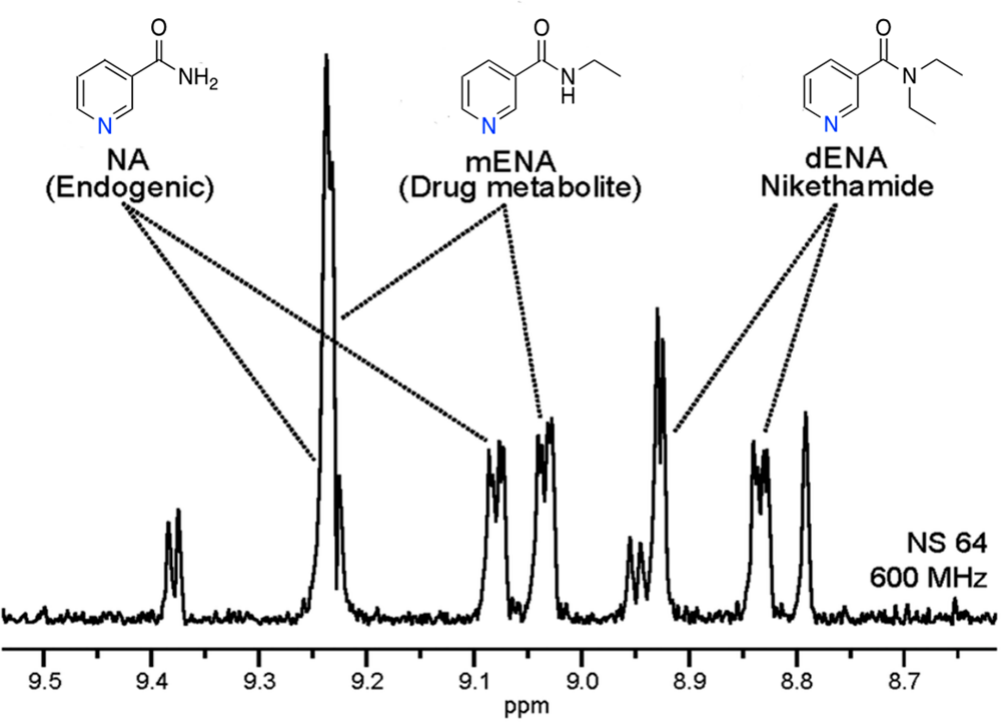
1D selective excitation spectrum of a urine SPE extract after spiking urine with 2 μM diethylnicotinamide (dENA, nikethamide) and ethylnicotinamide (mENA). Adapted with permission from ref (3). Copyright 2016 the authors. Published by Royal Society of Chemistry under a Creative Commons Attribution-NonCommercial 3.0 Unported License (https://creativecommons.org/licenses/by-nc/3.0/).

The structure of the iridium complex formed by the association of nicotinamide with the iridium catalyst is indicated together with the corresponding high-field hydride signal (Figure 12). By selective excitation, it is possible to transfer the enhanced magnetization of this hydride to the *ortho*-protons of nicotinamide. This approach has allowed selective NMR detection of the aromatic protons of nicotinamide and of a doping substance, nikethamide, down to sub-μM concentrations in urine (Figure 13).^3^ Additional nhPHIP studies in the chemical analysis of urine have also recently been reported.^51,52^

#### 3.2.4 Enhanced Dispersion of Signals by the Zero-Quantum (ZQ) Approach

**Figure 14:**
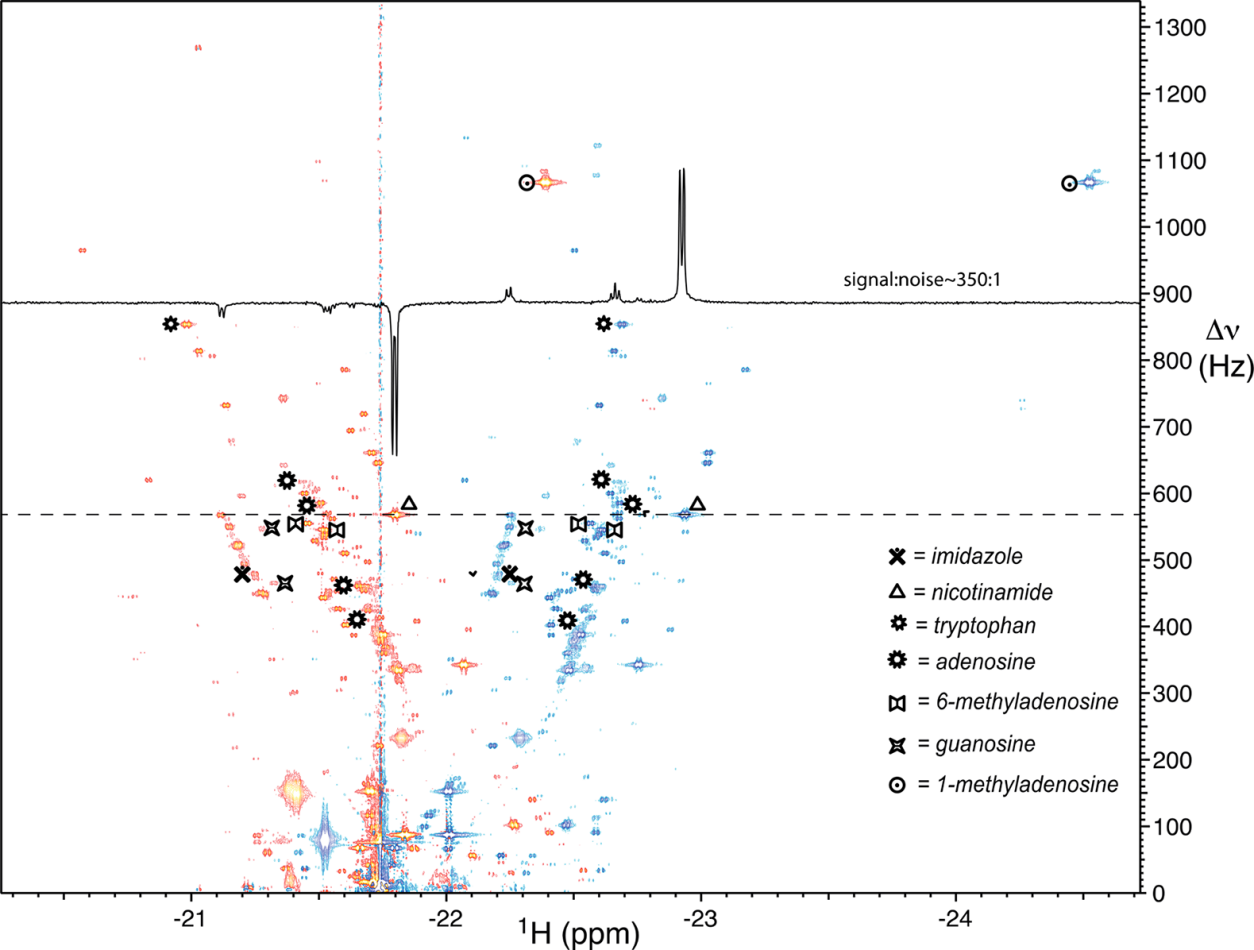
2D nhPHIP-ZQ hydrides spectrum for a solid phase extract of human urine in methanol-*d*_4_, with iridium catalyst (0.8 mM), mtz (15 mM), and 51%-enriched *p*-H_2_ (5 bar). The 1D trace displays the signals of the hydrides of nicotinamide. Adapted with permission from ref (4). Copyright 2019 the authors. Published by Royal Society of Chemistry under a Creative Commons Attribution-NonCommercial 3.0 Unported License (https://creativecommons.org/licenses/by-nc/3.0/).

The nhPHIP-NMR 1D hydride spectrum (Figure 12) is indicative of a wealth of urinary metabolites that can be detected with the iridium chemosensor. Typically, enhancement factors on the order of 10^3^ (e.g., 982-fold for nicotinamide, 1336-fold for methylpyrazine) were found for these urinary nitrogenous heteroaromatic metabolites. Spectral dispersion of these signals was achieved using a 2D nhPHIP zero-quantum (ZQ) experiment^4^ that, compared to the aforementioned 2D methods, presents the advantage of higher sensitivity and superior spectral resolution, as the evolution of ZQ coherence is not affected by the interhydride scalar coupling (*J* ≈ 7.5–9.0 Hz) and therefore no signal splitting is observed in the indirect dimension. The resolution of the hydride signals in the 2D nhPHIP-ZQ spectrum (Figure 14) was achieved by sampling the indirect dimension up to ca. 500 ms, which requires low dissociation rates of the nhPHIP-NMR chemosensor. This high stability seems to be a typical feature of the complexes formed using mtz as co-substrate^53,54^ and is crucial for nhPHIP-chemosensing applications in complex mixture analysis, in which high-resolution 2D NMR spectra are necessary to resolve extremely crowded regions.

In the 2D-nhPHIP ZQ spectrum (Figure 14) every pair of hydride signals of opposite sign (in blue and red) at the same frequency in the indirect dimension indicates a metabolite associating with the catalyst. On the basis of the number of signals observed in the ZQ spectrum, the number of compounds revealed by nhPHIP-NMR-chemosensing could be estimated at a few hundred. For some metabolites, multiple binding modes are possible (e.g., adenosine for which four structurally different complexes have been observed, giving four pairs of hydride signals in the spectrum).

Identification of the analytes associating with the iridium NMR chemosensor is a challenging task (see Section 2.3.). Signal assignments for seven analytes (Figure 14) were achieved by spiking the sample with known standards.

**Figure 15:**
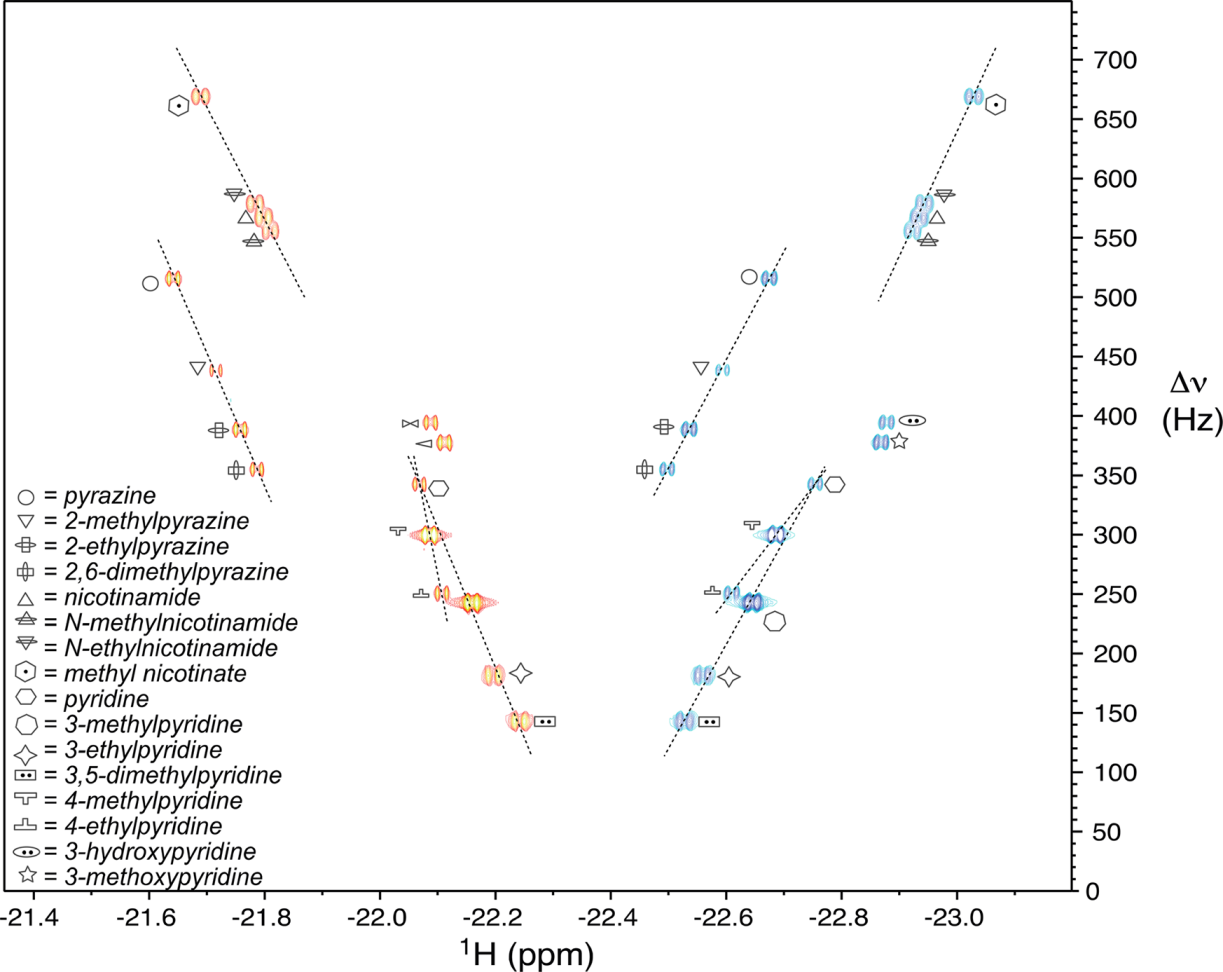
2D nhPHIP-ZQ spectrum of the hydrides for a number of iridium ligands in methanol-*d*_4_ in the presence of the iridium catalyst (0.8 mM), mtz (15 mM), and 51%-enriched *p*-H_2_ (5 bar). The signals of structurally homologous compounds are connected with dotted lines. Signal assignment is indicated. Adapted with permission from ref (4). Copyright 2019 the authors. Published by Royal Society of Chemistry under a Creative Commons Attribution-NonCommercial 3.0 Unported License https://creativecommons.org/licenses/by-nc/3.0/.

Interestingly, the 2D nhPHIP-ZQ spectrum of different series of homologous model substrates reveals linear patterns in the chemical shifts of the signals of the hydrides (Figure 15); different linear trends are clearly observed for 3- and 4-alkylated pyridines, alkylated pyrazines, and nicotinic acid derivatives. Such correlations can be helpful for the identification of the metabolites interacting with the iridium NMR chemosensor via the resonances of the hydrides, as shown in a recent study^55^ on the metabolism of nicotine in the urine of smokers. Because of the observed correlation, it was suspected that a third major analyte, next to nicotine and its major degradation product cotinine, was 3-hydroxycotinine, which was then confirmed by standard addition. With CDCl_3_ as an SPE eluent and NMR solvent, the relatively apolar nicotine and its metabolites were eluted with 95% recovery, and the NMR spectra were simplified because of the absence of more polar analytes. The limit of detection was found to be 0.1 μM, and that of quantitation, 0.7 μM.

The 2D nhPHIP-zero-quantum experiment has also been applied for the detection of α-amino acids at submicromolar concentrations in complex mixtures.^54^ α-Amino acids can bind to the iridium in the hyperpolarization catalyst as chelating ligands via the amino and carboxyl groups. The kinetically preferred mode of chelation, with both coordinating groups tightly bound to iridium in the equatorial plane, does not allow efficient *p*-H_2_ refreshment in the complex and is, consequently, PHIP-silent. The transition to the thermodynamically favored axial/equatorial chelation mode can be achieved, however, by heating to 50 °C for a few minutes. The rapid co-substrate (pyridine) exchange in the equatorial plane allows *p*-H_2_ refreshment in the complex and HF-nhPHIP signal enhancement for this binding mode. The approach was tested on a mixture of the 20 natural α-amino acids and sarcosine (*N*-methylglycine) at 1 μM concentration. The resonances of the hydrides were found to be highly sensitive to the amino acid side chains, giving sufficient dispersion of the signals for all components in the complex mixture to be distinguished; the combination of a chiral center in the amino acid with the stereogenic center on iridium in the complex leads to the formation of two diastereoisomeric complexes with different sets of NMR signals. Importantly, this approach can be directly applied to aqueous mixtures, such as biofluids, without any sample treatment, as demonstrated on a sample of human urine, in which natural α-amino acids could be measured after simple dilution with methanol.

### 3.3 Limitations

In previous sections, the application of nhPHIP and NMR chemosensing to the analysis of dilute components in complex mixtures has been discussed. Here, the main limitations of this approach are briefly summarized. In the first place, whereas the selectivity of nhPHIP represents the basis of the working principles of the NMR chemosensor, it actually excludes several classes of important metabolites/analytes from the scope of the technique. Analytes without coordination sites or molecules that coordinate too weakly to the nhPHIP catalyst cannot be detected with this approach. However, it should be mentioned that over the course of the past decade the number of compounds capable of associating with the iridium catalyst, and thus suitable for NMR chemosensing, has been continuously increasing. Second, although the NMR-chemosensing technique can be highly efficient in detecting, resolving, and quantifying dilute species in complex systems, the structural assignment of unknown compounds in the mixtures remains problematic. While elucidating the structures of unknown analytes cannot generally be achieved from the hydride resonances alone, compiling a database of hydride chemical shifts for known substrates can assist in the identification of unknown compounds from nhPHIP spectra. Third, the co-substrate mtz generally provides high stability to the catalytic complexes. Low dissociation rates are important for NMR chemosensing because they allow for the acquisition of high-resolution 1D and 2D nhPHIP spectra that are crucial when dealing with the signal crowding of complex systems. On the other hand, this high stability also determines a slow refreshment of *p*-H_2_ in the complex resulting in lower nhPHIP signal enhancements compared to other PHIP techniques. However, it should be noted that because nhPHIP methods are based on reversible interactions, repeated measurements on the same chemically unmodified substrate molecules are possible. This allows for the acquisition of multiple-scan 1D and 2D nhPHIP experiments, which can provide for a further increase in NMR sensitivity by signal averaging. In practice, a few minutes of acquisition, shortly bubbling *p*-H_2_ in the sample at the beginning of each scan, would result in a 10-fold increase in the signal-to-noise ratio.

## 4 Conclusions and Outlook

This Account has focused on the application of non-hydrogenative PHIP to analytes present in low concentrations in complex mixtures. We have shown that nhPHIP-NMR-chemosensing can be directly applied to biofluids or aqueous extracts with little to no sample manipulation, thereby significantly broadening the range of analytical problems to which NMR can be applied. Under the conditions illustrated here, the integrals of the hyperpolarized signals display a linear dependency on the analyte concentration, which is important for their quantification via calibration methods.

Even though there are still obstacles to solving the NMR sensitivity challenge, the use of nhPHIP techniques provides a giant leap forward in resolving these limitations. Catalyst development and technique development remain areas of focus, and currently agents exist that allow for the detection of a large variety of different compounds present in different solvent media. Although we have been able to circumvent the specific problems of PHIP in aqueous solutions—poorer solubility of catalysts and *p*-H_2_ in an aqueous solvent medium, lower exchange rates, and so forth—by applying solid-phase extraction or working in water–alcohol mixtures, it is worth noting that water-compatible catalysts have been developed. These catalysts widen the scope and applicability of PHIP techniques.

The identification of all analytes associating with the iridium NMR chemosensor is a challenging task, as the structural information is manifested only in the chemical shifts of the corresponding hydride pairs. Generally, signal assignment from the nhPHIP hydride spectra is achieved by tentatively spiking the samples. Interestingly, the 2D nhPHIP-ZQ hydride spectra display linear patterns in the position of the hydrides signals that seem to originate from catalyst binding of compounds with high structural homology. *Ab initio* chemical shift calculations might shed some light on these experimental observations and, it is hoped in the near future, assist the process of analyte identification from hyperpolarized hydrides spectra.
